# Meta-Analysis of the Association Between Asthma and the Risk of Stroke

**DOI:** 10.3389/fneur.2022.900438

**Published:** 2022-06-24

**Authors:** Zheng-Hua Fang, Zhi-Fei Li, Zhuo-Yu An, Si-Cheng Huang, Meng-Di Hao, Wei-Xing Zhang

**Affiliations:** ^1^The First People's Hospital of Jiande, Hangzhou, China; ^2^Aerospace Center Hospital, Beijing, China; ^3^Peking University People's Hospital, Beijing, China; ^4^Department of Respiratory Medicine, The First People's Hospital of Jiande, Hangzhou, China

**Keywords:** asthma, the risk of stroke, morbidity, meta-analysis, different population groups

## Abstract

**Introduction:**

Asthma and stroke share many risk factors. Previous meta-analysis has indicated that asthma is associated with an increased risk of stroke. However, this study were limited by the small number of articles included and the lack of subgroup analyses of different stroke types and different populations. This meta-analysis aimed to synthesize evidence systematically to investigate the impact of asthma on stroke.

**Methods:**

We searched Medline (*via* PubMed), Web of Science and EMBASE databases and manually identified eligible studies (inception dates to December 25, 2021) that analyzed the association between asthma and stroke. We conducted quality assessment to evaluate the risk of bias of studies and sensitivity analyses to test the robustness of results.

**Results:**

We included 8 cohort studies and 10 cross-sectional studies comprised 3,011,016 participants. We found patients with asthma had a higher risk of stroke than patients without asthma [relative risk (RR): 1.34, 95% confidence interval (CI): 1.21–1.47]. Moreover, asthma significantly increased the risk of ischemic stroke (RR: 1.25, 95% CI: 1.06–1.47) without increasing the risk of hemorrhagic stroke (RR: 1.08, 95% CI: 0.87–1.34). Asthma increased the risk of stroke in both men (RR: 1.20, 95% CI: 1.10–1.32) and women (RR: 1.29, 95% CI: 1.12–1.48) with no significant difference between the sexes. We also found that patients with inactive asthma, child-onset asthma, or no smoking history did not have an increased risk of stroke.

**Conclusions:**

These results supported the finding that asthma could significantly increase the risk of stroke, but this impact was not consistent in different populations.

**Systematic Review Registration:**

https://www.crd.york.ac.uk/PROSPERO/display_record.php?RecordID=290745, identifier: CRD42021290745.

## Introduction

Asthma is a common chronic inflammatory disease. Globally, it is estimated that 334 million people have asthma, including children and adults (1). The incidence of asthma continues to increase, particularly in places with a western lifestyle (2), which aggravates the health and financial burden of society.

Patients with asthma have reduced lung function and a high risk of comorbidities. Essentially, asthma is due to chronic airway inflammation, and the main features are airway hyper-reactivity and reversible airway limitation. However, chronic asthmatic inflammation is not limited to the airway, but affects the whole body (3, 4). Consequently, asthma is a risk factor for stroke (5). In addition, asthma and stroke share other risk factors, including smoking and obesity (6, 7).

Stroke is responsible for over 5.5 million deaths annually, and it is estimated that 166 million healthy-life years are lost due to stroke (8). The question of whether asthma increases the risk of stroke is currently unresolved, and it has become a hot topic at the intersection of respiratory medicine and neurology (9, 10).

Considering the growing incidence of asthma and the high disability rate and mortality associated with stroke, there is an urgent need to determine how asthma affects the risk of stroke. Moreover, knowledge about the degree of stroke risk induced by asthma in different populations will assist in developing population-wide interventions. In addition, this knowledge could inform the clinical management of patients with asthma that have high stroke risk.

A previous meta-analysis of 5 cohort studies noted that asthma was associated with an increased risk of stroke (11). However, due to insufficient data in the studies included, that review failed to analyze the stroke risk in different populations with asthma. To avoid these limitations and provide reliable evidence, we conducted a meta-analysis that included cohort studies and cross-sectional studies to investigate the impact of asthma on stroke.

## Materials and Methods

This meta-analysis was conducted according the MOOSE (Meta-Analysis of Observational Studies in Epidemiology) and PRISMA (Preferred Reporting Items for Systematic Reviews and Meta-Analyses) checklist (Supplementary Table 1), to ensure quality (12). This meta-analysis was previously registered on the International Prospective Registry of Systematic Reviews (PROSPERO Identifier: CRD 42021290745).

### Literature Search

We searched electronic databases up to December 25, 2021 (PubMed, EMBASE). The detailed search terms are provided in Supplementary Table 2. In addition, to ensure the adequacy of the included studies, we conducted a manual search.

### Selection Criteria and Outcomes of Interest

We included observational cohort studies with both prospective and retrospective designs. The inclusion criteria were: both asthma and control groups had to be included; the total number of subjects had to be >1,000, to reduce bias; outcome measures had to include an analysis of the association between asthma and stroke; and the data had to include hazard ratios (HRs), relative risks (RRs), odds ratios (ORs), or the number of stroke events in the asthma and control groups.

### Data Abstraction

The titles and abstracts of eligible studies were analyzed independently by two independent investigators (Z-FL and Z-YA). Disagreements were settled by discussing with a third author (Z-HL) to reach a consensus. Then, the reviewers independently assessed the full text of all studies for inclusion, based on the criteria related to population selection, study design, and outcome. The investigators also attempted to contact the corresponding author for information on missing data.

### Assessment of the Risk of Bias

The risk of study bias and quality were assessed with the Newcastle-Ottawa Scale (NOS) for cohort studies. NOS scores below 6 indicated low quality, NOS scores of 6 or 7 indicated medium quality, and NOS scores above 7 indicated high quality. Two authors (S-CH and M-DH) independently completed the quality assessments. In cases of disagreement, a third investigator (Z-FL facilitated the discussion and made the final decision).

### Data Analysis

We pooled the data and calculated adjusted HRs, RRs, or ORs with 95% confidence intervals (95% CI). When adjusted estimates were unavailable, we included unadjusted estimates and the number of events to calculate stroke incidence in the asthma and control groups. The *I*^2^ statistic was used to evaluate the heterogeneity of the included studies. To address the variability of each outcome of interest, an *I*^2^ > 50% was defined as significant heterogeneity (13), and we used the random-effects model. Data were analyzed with Stata version 14.0 (Stata Corp., College Station, TX) or Review Manager 5.3 software (2014; The Nordic Cochrane Center, The Cochrane Collaboration, Copenhagen, Denmark).

### Additional Analyses

We conducted subgroup analyses investigating the risk of stroke of people with asthma in different stroke types (including ischemic stroke and hemorrhagic stroke) and different populations including asthma status, smoking status, gender and age of asthma onset. We also conducted sensitivity analyses to test the robustness of results.

## Results

**Figure 1:**
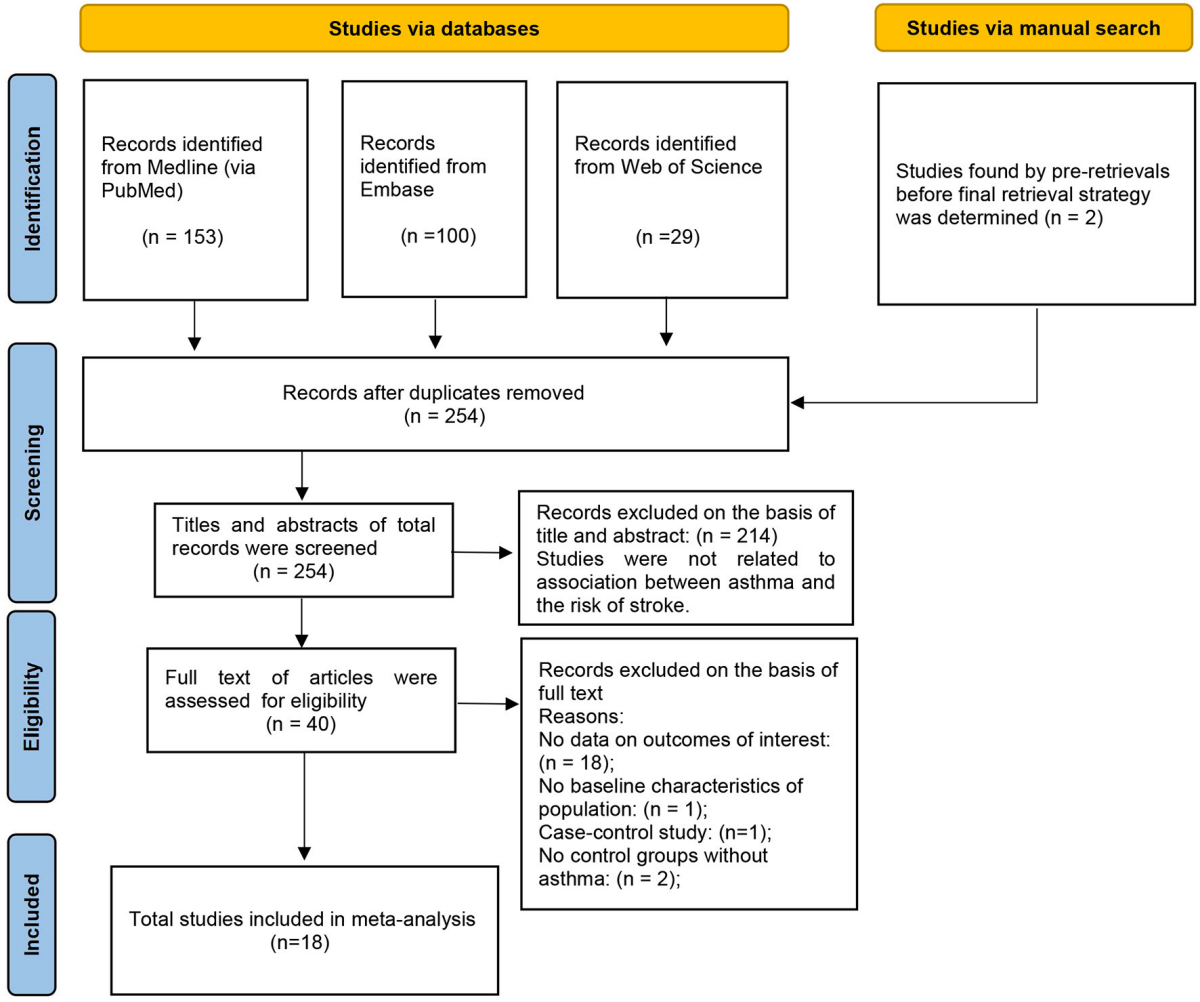
Prisma flow chart of article selection process.

Our searches identified 254 articles. After applying the inclusion and exclusion criteria, we included 18 observational studies (9, 14–30) (Figure 1). Among these, 2 studies (21, 22) were identified in the manual search and 16 studies (9, 14–20, 23–30) were identified in the electronic search.

### Description of Studies

Among 18 the observational studies included, 10 were cross-sectional studies (21–30). Of these, 2 were cohort studies (21, 28) that did not have outcomes of interest, but reported cross-sectional data on the association between asthma and stroke in the baseline characteristics of the population. The other 8 cross-sectional cohort studies (9, 14–20) included 2 retrospective studies (17, 19) and 6 prospective studies (9, 14–16, 18, 20).

We collected baseline patient characteristics from each study (Table 1). The combined populations of these studies yielded a total of 3,011,016 participants. Six studies were conducted in Asia, 6 were conducted in North America, 4 were conducted in Europe, and 2 were conducted in Oceania. The quality assessments with the NOS (Supplementary Table 3) showed that 4 studies were high quality, 10 were medium quality, and 4 were low quality. Six studies presented data about the risk of ischemic stroke in patients with asthma, and 3 studies presented data about the risk of hemorrhagic stroke in patients with asthma. Some studies examined the impact of asthma on stroke in different subgroups, based on sex, smoking status, asthma status, and age of asthma onset.

**Table 1:** Baseline characteristics of participants assessed in the studies included in the meta-analysis.

**References**	**Total**									**Confounding factors adjusted for in the analyses**	**NOS score**
	**Population**	**Participant number**	**Study Type**	**Follow-up, mean or (range)**	**Age, range or (mean ±SD)**	**Gender (female, %)**	**Diagnosis criteria of asthma**	**Diagnosis criteria of stroke**	**Comorbidities, *n* (%)**		
Onufrak et al. (15)	Community	14,567	Cohort study, prospective	12–14	45–64	56.9%	Self-reported	Doctor diagnosis	DM:10.9%, HTN:33.5%, CB:8.4%, emphysema:1.6%	NA	7
Chung et al. (17)	Community	72,587	Cohort study, retrospective	7.2	52.1 ± 17.6	54.3%	Electronic medical record	Electronic medical record	AF:0.5%, HTN:29.1%, HPL: 16.2%, HF:2.0%, COPD: 9.9%, DVT:0.1%, CHD:13.7%	Age, sex and comorbidities of AF, HTN, HPL, HF, alcoholism, obesity, COPD, DVT and CHD	7
Çolak et al. (18)	Community	94,079	Cohort study, prospective	<9.4	56.1	59.2%	Doctor diagnosis	Doctor diagnosis	Pneumonia or acute bronchitis:22.5%	Age, sex, BMI, leisure time physical activity, education, annual household income, alcohol, cumulative tobacco consumption, BP, TC, LDL-C, HDL-C, TG, use of cholesterol-lowering medication, and DM	7
Kim et al. (19)	Community	234,728	Cohort study, retrospective	<12	>20	63.7%	Electronic medical record	Electronic medical record	HTN:38.8%, DM:20%, IHD:6.8%, depression:10.4%, dyslipidemia:28.2%	Age, sex, income, region of residence, HTN, DM, HPL, IHD, depression histories	8
Cepelis et al. (16)	Community	57,104	Cohort study, prospective	17.2 ± 5.4	>20	52.9%	Self-reported	Electronic medical record	DM:2.2%, HTN:37.5%	Age, birth year cohort, sex, BMI, smoking status, alcohol use, education level, TC/HDL ratio, HTN, DM	8
Iribarren et al. (20)	Community	407,190	Cohort study, prospective	2.3–9.3	≥18	66.0%	Electronic medical record	Electronic medical record	HTN:7.2%, DM:5.6%, HPL:6.1%	DM, HTN, HPL, BMI, smoking status and history of any allergy	7
Schanen et al. (14)	Community	13,501	Cohort study, prospective	≥14	54 ± 5.9	57.0%	Self-reported	Electronic medical record	DM:10.7%, HTN:23.5%	Age, sex, race/center, HDL-C, LDL-C, BP, HTN medication use, smoking status, pack years, WHR, DM, and sport score	8
Tattersal et al. (9)	Community	6,792	Cohort study, prospective	9.1 ± 2.8	62.1 ± 10.2	52.9%	Self-reported	Electronic medical record	DM:12.6%	Age, race, sex, TC, HDL-C, BP, smoking, DM, anti-HTN and lipid-lowering medication use, BMI, family history of CVD, income	8
Wee et al. (29)	Community	162,570	Cross-sectional study		53.3 ± 8.4	65.7%	Self-reported	Electronic medical record	HTN:22.5%, DM:8.0%, HPL:13.4%, IHD:3.1%	Age, sex, income, BMI, smoking, alcohol, HTN,DM,HPL, other allergic rhinitis histories, nutritional intake	7
Enright et al. (22)	Community	5,169	Cross-sectional study		72.8 ± 5.6	57.0%	Self-reported	Electronic medical record	NA	NA	5
Lee et al. (24)	Community	16,943	Cross-sectional study		49.5 ± 10.0	51.7%	Self-reported	Doctor diagnosis	DM:7.7%, MI:3.2%, CHD:5.6%	Age, BP, HDL-C, BMI, Hs-CRP, smoking, and DM	6
Adams et al. (30)	Community	7,443	Cross-sectional study		≥18	49.1%	Self-reported	Self-reported	NA	Age and sex	5
Appleton et al. (23)	Community	4,060	Cross-sectional study		≥18	NA	Doctor diagnosis	Self-reported	DM:13.0%, CVD:12.3%, Metabolic syndrome:45.7%	Age (>50 years), sex, smoking, MI	6
Bozek et al. (26)	Community	2,099	Cross-sectional study		67.9 ± 5.6	53.6%	Doctor diagnosis	Electronic medical record	HTN:44.5%, HPL:48.0%, CHD:23.6%, arrhythmias:2.6%, HF:18.6%, hyperuricemia:6.0%, dementia: 4.1%, depression: 17.5%, delirium:4.1%, anxiety:8.9%	NA	5
Park et al. (25)	Community	4,445	Cross-sectional study		46–68	58.4%	Electronic medical record	Self-reported	Arthritis:28.6%, HTN:37.7%, Dyslipidemia:32.7%, DM:12.6%, Depression:16.6%, Obesity:11.9%	NA	6
Weatherburn et al. (27)	Community	1,424,378	Cross-sectional study		≥18	50.9%	Electronic medical record	Electronic medical record	COPD:3.7%, HF:1.3%, CHD:5.7%, DM:5.3%, HTN:16.5%, AF:15.7%	Age, sex, deprivation, smoking	6
Strand et al. (28)	Community	446,346	Cross-sectional study		40.0 ± 13.5	50.9%	Self-reported	Self-reported	DM:8.8%, CHD:3.3%, cancer:9.3%, arthritis:24.1%	NA	5
He et al. (21)	Community	37,015	Cross-sectional study		≥20	51.9%	Self-reported	Electronic medical record	DM:8.8%, CHD:3.3%, cancer:9.3%, arthritis: 24.1%	NA	6

*AF, atrial fibrillation; BMI, body mass index; BP, blood pressure; CB, chronic bronchitis; CVD, cardiovascular disease; CHD, coronary heart disease; DM, diabetes mellitus; DVT, deep vein thrombosis; HDL, high-density lipoprotein; HDL-C, high-density lipoprotein cholesterol; HPL, hyperlipidemia; HTN, hypertension; HF, heart failure; LDL, low-density lipoprotein; LDL-C, low-density lipoprotein cholesterol; MI, myocardial infarction; NA, not available; TC, total cholesterol; TG, triglycerides; WHR, waist-hip ratio*.

### Association Between Asthma and Stroke

**Figure 2:**
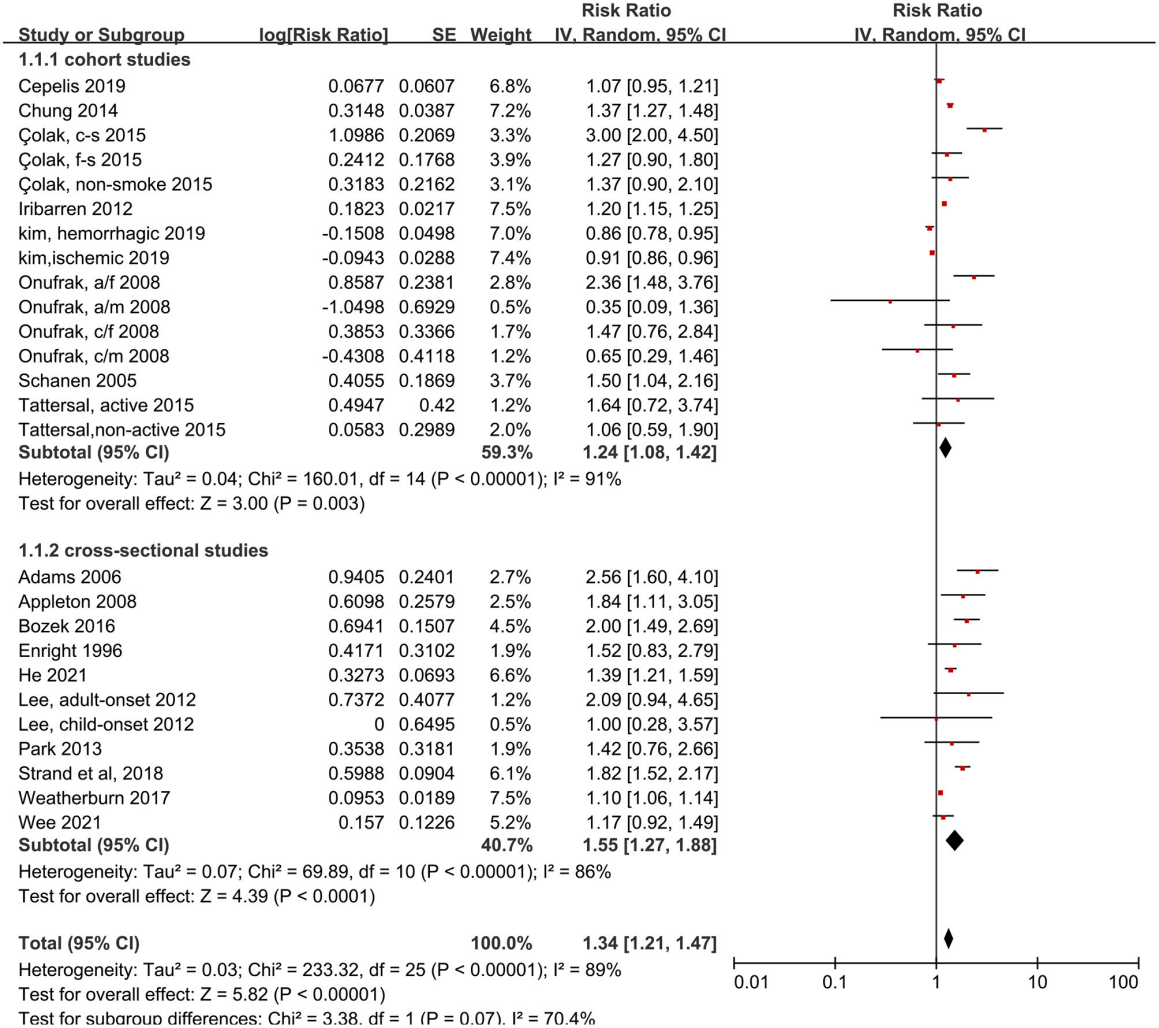
Forest plot shows association between asthma and risk of stroke. SE, standard error; IV, Inverse Variance method; df, degrees of freedom; CI, confidential interval; a/f, adult female; a/m, adult male; c/f, child female; c/m, child male; c-s, current smoking; f-s, former smoking; n-s, never smoking.

We analyzed 18 studies and 26 subsets with a random-effects model. That analysis revealed that patients with asthma had a 34% higher risk of stroke than individuals without asthma (RR: 1.34, 95% CI: 1.21–1.47, *p* < 0.00001; *I*^2^: 89%, *p* < 0.00001). Most of the subsets (22 of 26) showed an increased risk of stroke in patients with asthma, but the heterogeneity was pronounced. Moreover, the risk of stroke was significantly increased with asthma, in both the cohort studies (RR: 1.24, 95% CI: 1.08–1.42, *p* = 0.003) and the cross-sectional studies (RR: 1.55, 95% CI: 1.27–1.88, *p* < 0.00001), and no difference was found between analysis results of cohort studies and cross-sectional studies (*p* = 0.07 for between-group difference) (Figure 2).

**Figure 3:**
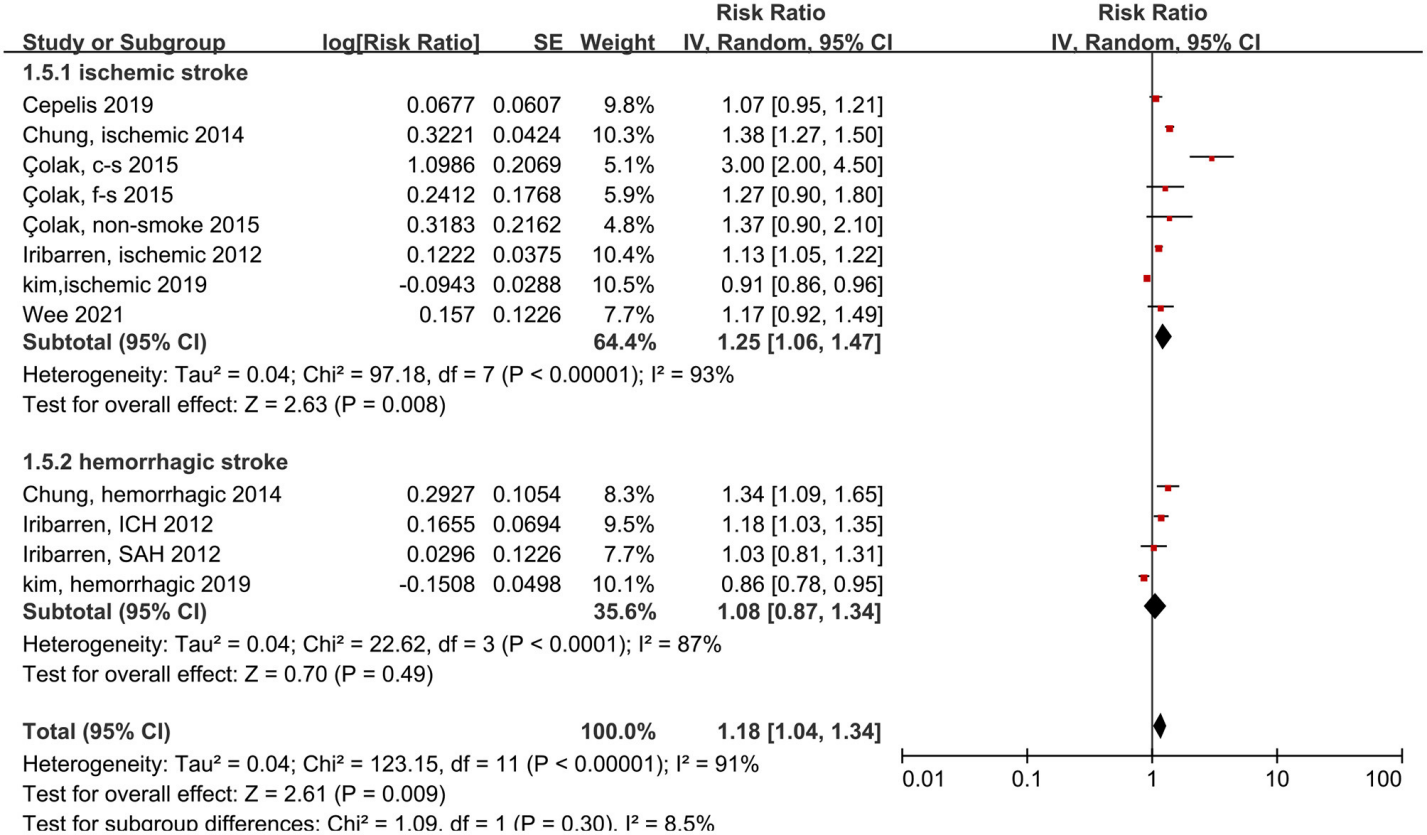
Forest plot shows association between asthma and the risk of ischemic or hemorrhagic stroke. SE, standard error; IV, Inverse Variance method; df, degrees of freedom; c-s, current smoking; f-s, former smoking; ICH, intracerebral hemorrhage; SAH, Subarachnoid hemorrhage.

We also measured the effects of asthma on ischemic and hemorrhagic stroke. Patients with asthma had 25% more risk of ischemic stroke compared to people without asthma, and this difference was significant (RR: 1.25, 95% CI: 1.06–1.47, *p* < 0.00001; *I*^2^: 93%, *p* < 0.00001). In contrast, the probability of hemorrhagic stroke did not increase significantly in patients with asthma (RR: 1.08, 95% CI: 0.87–1.34, *p* = 0.49; *I*^2^: 87%, *p* < 0.0001) (Figure 3).

### Association Between Asthma and Stroke in Different Populations

**Figure 4:**
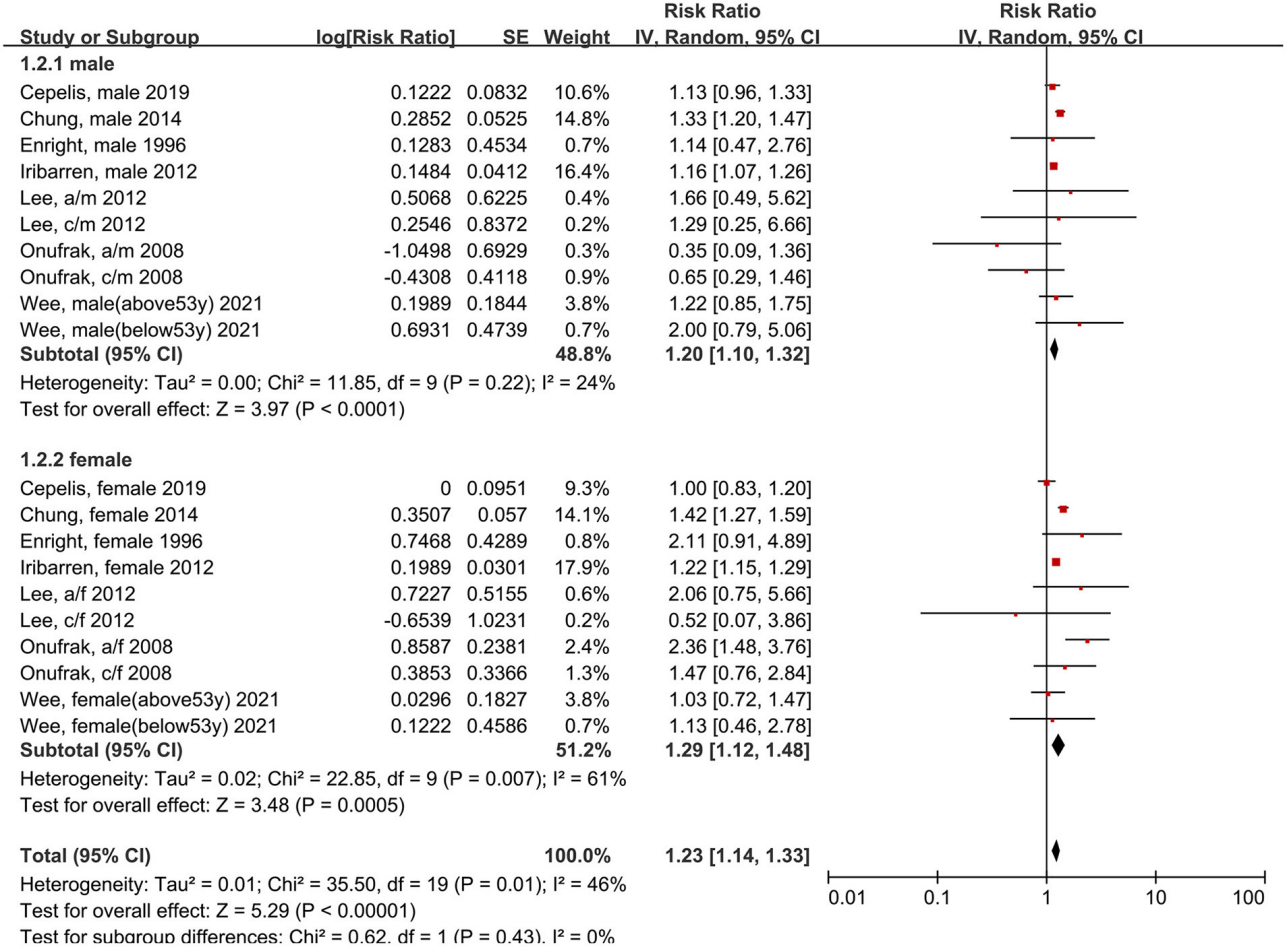
Forest plot shows association between asthma and the risk of stroke in male or female. SE, standard error; IV, Inverse Variance method; df, degrees of freedom; a/f, adult female; a/m, adult male; c/f, child female; c/m, child male.

Next, we examined the risk of stroke in different populations. The risk of stroke was significantly increased in both men (RR: 1.20, 95% CI: 1.10–1.32, *p* < 0.0001; *I*^2^: 24%, *p* = 0.22) and women (RR: 1.29, 95% CI: 1.12–1.48, *p* = 0.0005; *I*^2^: 61%, *p* = 0.007) with asthma, compared to individuals without asthma (Figure 4). However, no significant difference was found between the sexes (*p* = 0.07 for between-group differences).

**Figure 5:**
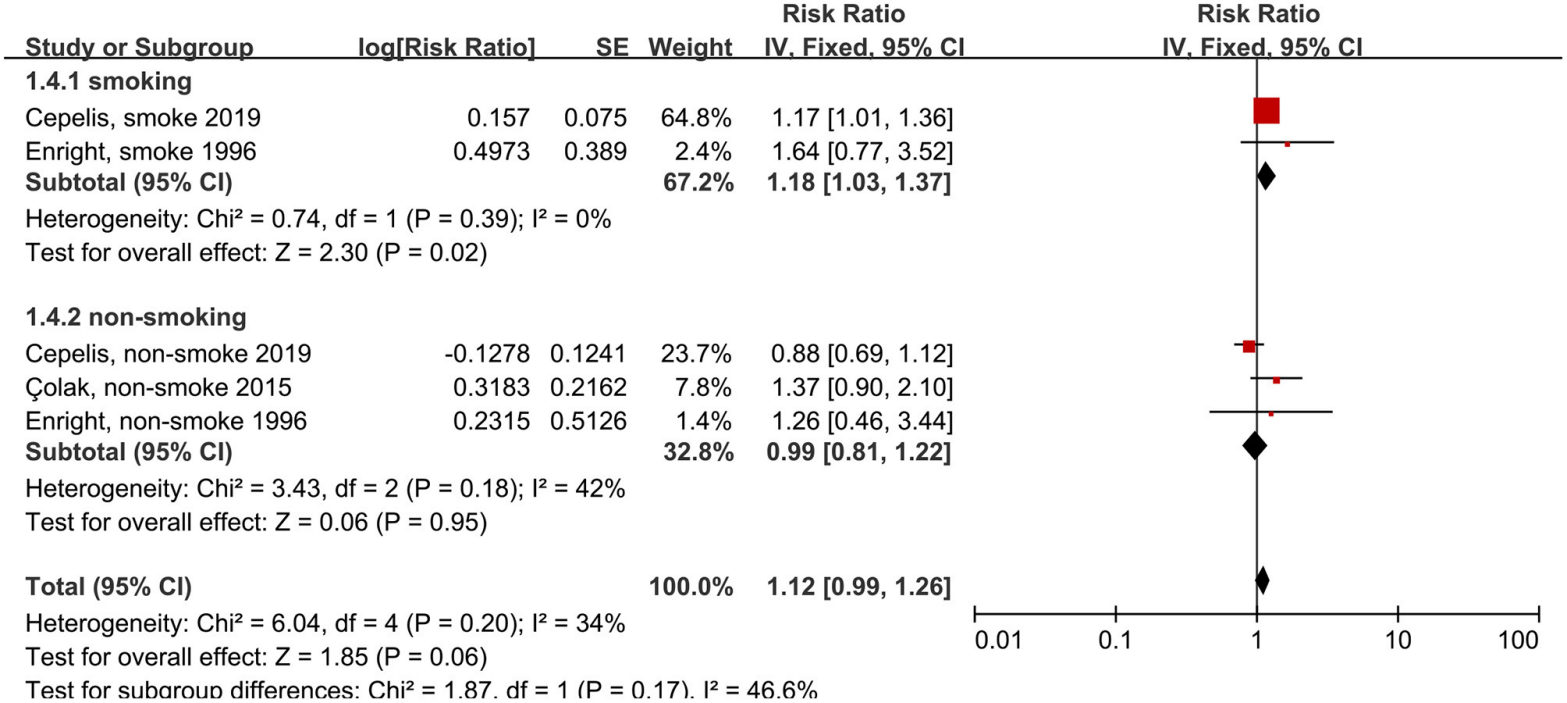
Forest plot shows association between asthma and the risk of stroke in smoking or non-smoking people. SE, standard error; IV, Inverse Variance method; df, degrees of freedom.

We also analyzed whether smoking status influenced the association between asthma and stroke risk. The subgroups of smoking included ever smokers (those that had smoked in the past or were smoking currently), and non-smokers (those that had never smoked). The reference subgroups were individuals with the same smoking status, but without asthma. We found that, among patients that did not have history of smoking, those with asthma did not have an elevated risk of stroke, compared to those without asthma (RR: 1.18, 95% CI: 1.03–1.37, *p* = 0.02; *I*^2^: 0%, *p* = 0.39). Conversely, we found that, among patients with a smoking history, those with asthma had an elevated risk of stroke, compared to those without asthma (RR: 0.99, 95% CI: 0.81–1.22, *p* = 0.95; *I*^2^: 42%, *p* = 0.18; Figure 5).

**Figure 6:**
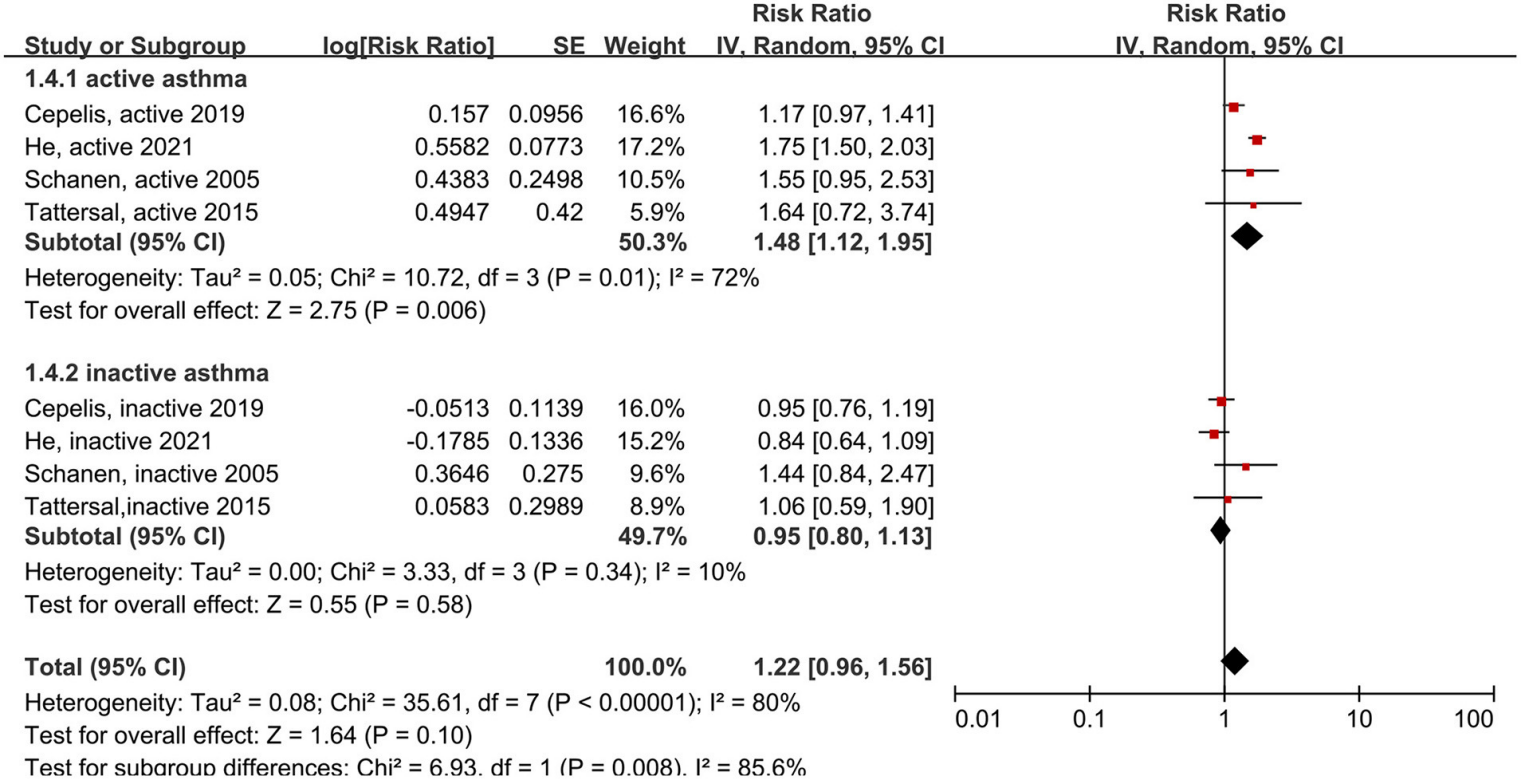
Forest plot shows association between different asthma status (including active asthma and inactive asthma) and the risk of stroke. SE, standard error; IV, Inverse Variance method; df, degrees of freedom.

Next, we compared patients with active asthma to patients with inactive asthma. Active asthma was defined as either a negative response to the question, “Do you still have asthma?” (14, 21), or evidence that the patient had not recently used asthma medication (9, 16). The risk of stroke was elevated in patients with active asthma (RR: 1.48, 95% CI: 1.12–1.95, *p* = 0.006; *I*^2^: 72%, *p* = 0.01), but not in patients with inactive asthma (RR: 0.95, 95% CI: 0.80–1.13, *p* = 0.58; *I*^2^: 10%, *p* = 0.34) (Figure 6). Thus, asthma activity had a significant effect on the association between asthma and stroke (*p* = 0.008 for the between-group difference).

**Figure 7:**
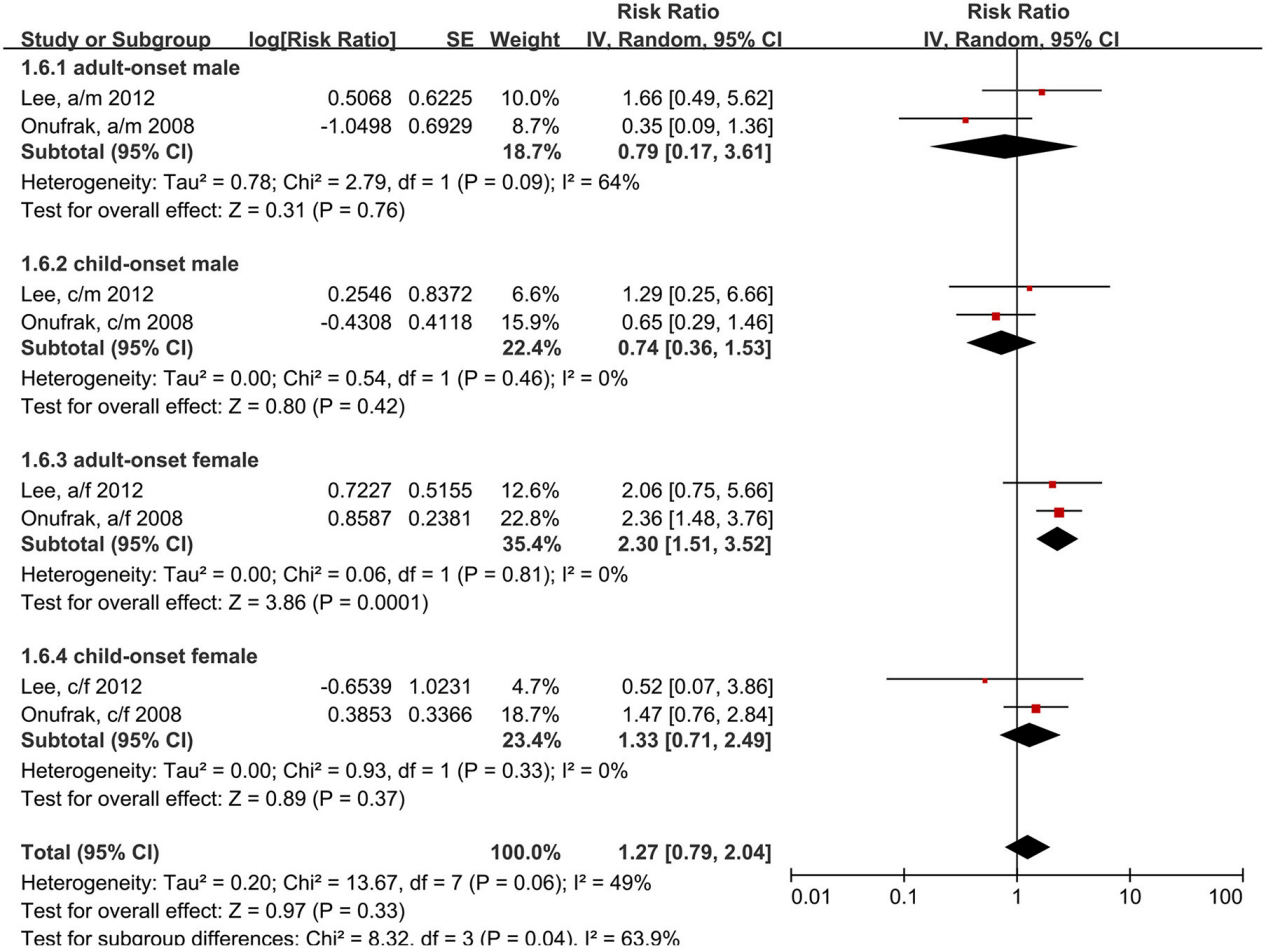
Forest plot shows association between asthma and the risk of stroke in different age of asthma onset and different gender. SE, standard error; IV, Inverse Variance method; df, degrees of freedom; a/f, adult female; a/m, adult male; c/f, child female; c/m, child male.

We also analyzed the age of asthma onset and patient sex. Child-onset and adult-onset asthma were defined by the age of the first asthma onset. In two studies (15, 24), the cut-off ages were 18 and 21 years. The results showed that only women with adult-onset asthma had an increased risk of stroke, compared to individuals without asthma (RR: 2.30, 95% CI: 1.51–3.52, *p* = 0.001; *I*^2^: 0%, *p* = 0.81) (Figure 7).

### Sensitivity Analyses

We performed sensitivity analyses to test the robustness of the main outcomes (Supplementary Figures 1–5). By omitting one study at a time and re-computing the pooled effect estimates, we did not find any significant difference in the results. We also analyzed the data with studies which did not adjust for blood lipid levels (or hyperlipidemia), blood pressure levels (or hypertension), or diabetes mellitus were omitted, and the result was robust (Supplementary Figure 6). Two studies with only an association between asthma and stroke but not have the study outcomes and 4 low quailty studies might reduce the quality of the data. We analyzed the data with these studies omitted and the result was robust (Supplementary Figure 7).

### Publication Bias

The publication bias was evaluated with a funnel plot. The funnel plot was visually symmetrical overall (Supplementary Figure 8).

## Discussion

This study assessed the effects of asthma on stroke by including previous studies comprehensively, particularly those with data for different populations. Our results showed that patients with asthma had a 34% higher risk of stroke than those without asthma. Furthermore, active asthma significantly increased the risk of stroke compared to inactive asthma (RR: 1.48 vs. RR: 0.95, *p* = 0.008). These results were consistent with previous studies, which showed that asthma, particularly active asthma, significantly increased the risk of stroke (11).

The chronic inflammatory state of asthma accelerates the process of atherosclerosis, which can lead to the development of stroke (9). Asthma and stroke share many common risk factors, including advanced age, smoking, obesity, air pollution, and stress. Thus, the association between asthma and stroke is, to some extent, affected by confounding factors (18, 31). Previous studies have pointed out that asthma was an independent risk factor for stroke. Asthma induced endothelial damage, accelerated atherosclerosis progression, and even caused thrombosis, due to decreased lung function and hypoxemia (4, 29). Previous studies have shown that elevated eosinophils (32), C-reactive protein (33), eosinophil cationic protein (34), and eotaxin (35) were involved in the development of atherosclerosis in patients with asthma. These features might explain why active asthma increases the risk of stroke: in active asthma, inflammation is severe and hypoxemia is prolonged, and these factors accelerate the atherosclerotic process.

Previous studies have suggested that asthma caused similar increases in the risks of ischemic and hemorrhagic stroke. In contrast, the present study showed that asthma mainly increased the risk of ischemic stroke and had less effect on the risk of hemorrhagic stroke. Our findings were supported by the association between asthma and atherosclerosis. Indeed, atherosclerosis directly drives thrombosis, which leads to ischemic stroke, and it is much less related to hemorrhagic stroke. In contrast, the main risk factors for hemorrhagic stroke are trauma and congenital vascular developmental malformations, which are more weakly associated with asthma.

The greatest strength of this study was that it included the largest sample size of any study to date that aimed to analyze the relationship between asthma and stroke. Moreover, we characterized the effect of smoking status on this association with a sufficiently sized subgroup analysis.

### Limitations

However, this study also had some limitations. First, unfortunately, we could not obtain sufficient data on patient asthma treatments to analyze their effects on stroke risk. Previous studies have shown that asthma treatment medications, like β receptor-agonists and glucocorticoids, could influence stroke outcomes (36–39). Moreover, we only included observational studies; therefore, we could not infer any direct causal relationships. Additionally, observational studies are susceptible to confounding factors that may bias the results. Finally, the heterogeneity of the included studies was not negligible; therefore, our results should be interpreted with caution.

### Conclusions

In conclusion, this meta-analysis provided evidence to support the notion that asthma is an important risk factor for stroke. This information is important for the development of strategies for preventing and treating asthma and stroke. In the future, well-designed, large, multicenter cohort studies should be conducted with a particular focus on a quantitative assessment of the effects of asthma on patients during stroke treatment.

## Data Availability Statement

The original contributions presented in the study are included in the article/Supplementary Material, further inquiries can be directed to the corresponding author/s.

## Author Contributions

Z-FL: analysis of data, interpretation results, drafting, and editing the article. Z-HF, Z-YA, S-CH, M-DH, and W-XZ: design of study, solution of problem, and reviewing the article. S-CH and M-DH: extraction of data and assessment of articles quality. All authors contributed to the article and approved this version of article.

## Conflict of Interest

The authors declare that the research was conducted in the absence of any commercial or financial relationships that could be construed as a potential conflict of interest.

## Publisher's Note

All claims expressed in this article are solely those of the authors and do not necessarily represent those of their affiliated organizations, or those of the publisher, the editors and the reviewers. Any product that may be evaluated in this article, or claim that may be made by its manufacturer, is not guaranteed or endorsed by the publisher.

## Supplementary Material

Supplementary Figure 1 Sensitivity analysis of association between asthma and the risk of stroke. CI, confidential interval; a/f, adult female; a/m, adult male; c/f, child female; c/m, child male; c-s, current smoking; f-s, former smoking; n-s, never smoking.

Supplementary Figure 2 Sensitivity analysis of association between asthma and the risk of stroke (only cohort studies included). CI, confidential interval; a/f, adult female; a/m, adult male; c/f, child female; c/m, child male; c-s, current smoking; f-s, former smoking; n-s, never smoking.

Supplementary Figure 3 Sensitivity analysis of association between asthma and the risk of stroke (only cross-sectional studies included). CI, confidential interval.

Supplementary Figure 4 Sensitivity analysis of association between asthma and the risk of stroke in male. CI, confidential interval; a/m, adult male; c/m, child male.

Supplementary Figure 5 Sensitivity analysis of association between asthma and the risk of stroke in female. CI, confidential interval; a/f, adult female; c/f, child female.

Supplementary Figure 6 Association between asthma and the risk of stroke (studies which did not adjust blood lipid, blood pressure, or diabetes mellitus were omitted). SE, standard error; IV, Inverse Variance method; df, degrees of freedom; CI, confidential interval; a/f, adult female; a/m, adult male; c/f, child female; c/m, child male; c-s, current smoking; f-s, former smoking; n-s, never smoking.

Supplementary Figure 7 Association between asthma and the risk of stroke (Two studies with only an association between asthma and stroke but not have the study outcomes and 4 low quailty studies were omitted). SE, standard error; IV, Inverse Variance method; df, degrees of freedom; CI, confidential interval; a/f, adult female; a/m, adult male; c/f, child female; c/m, child male; c-s, current smoking; f-s, former smoking; n-s, never smoking.

Supplementary Figure 8 Funnel plot depicting publication bias for the association between asthma and the risk of stroke. SE, standard error; RR, risk ratio.

Supplementary Table 1 PRISMA 2009 checklist.

Supplementary Table 2 Search strategies.

Supplementary Table 3 Newcastle-Ottawa scale for assessing the quality of included studies.

## Abbreviations

CI: confidential interval
ORs: odds ratios
RR: relative risk
HRs: hazard ratios
NOS: Newcastle-Ottawa Scale
PRISMA: Preferred Reporting Items for Systematic Reviews and Meta-Analyses
MOOSE: Meta-Analysis of Observational Studies in Epidemiology
PROSPERO: Prospective Register of Systematic Reviews
SE: standard error
IV: Inverse Variance method
df: degrees of freedom
a/f: adult female
a/m: adult male
c/f: child female
c/m: child male
c-s: current smoking
f-s: former smoking
n-s: never smoking.
