# Supplementary figures and images for: Meta-Analysis of the Association Between Asthma and the Risk of Stroke
Source: Front Neurol. 2022 Jun 24;13:900438. doi: 10.3389/fneur.2022.900438 (PMC9263265; doi:10.3389/fneur.2022.900438)

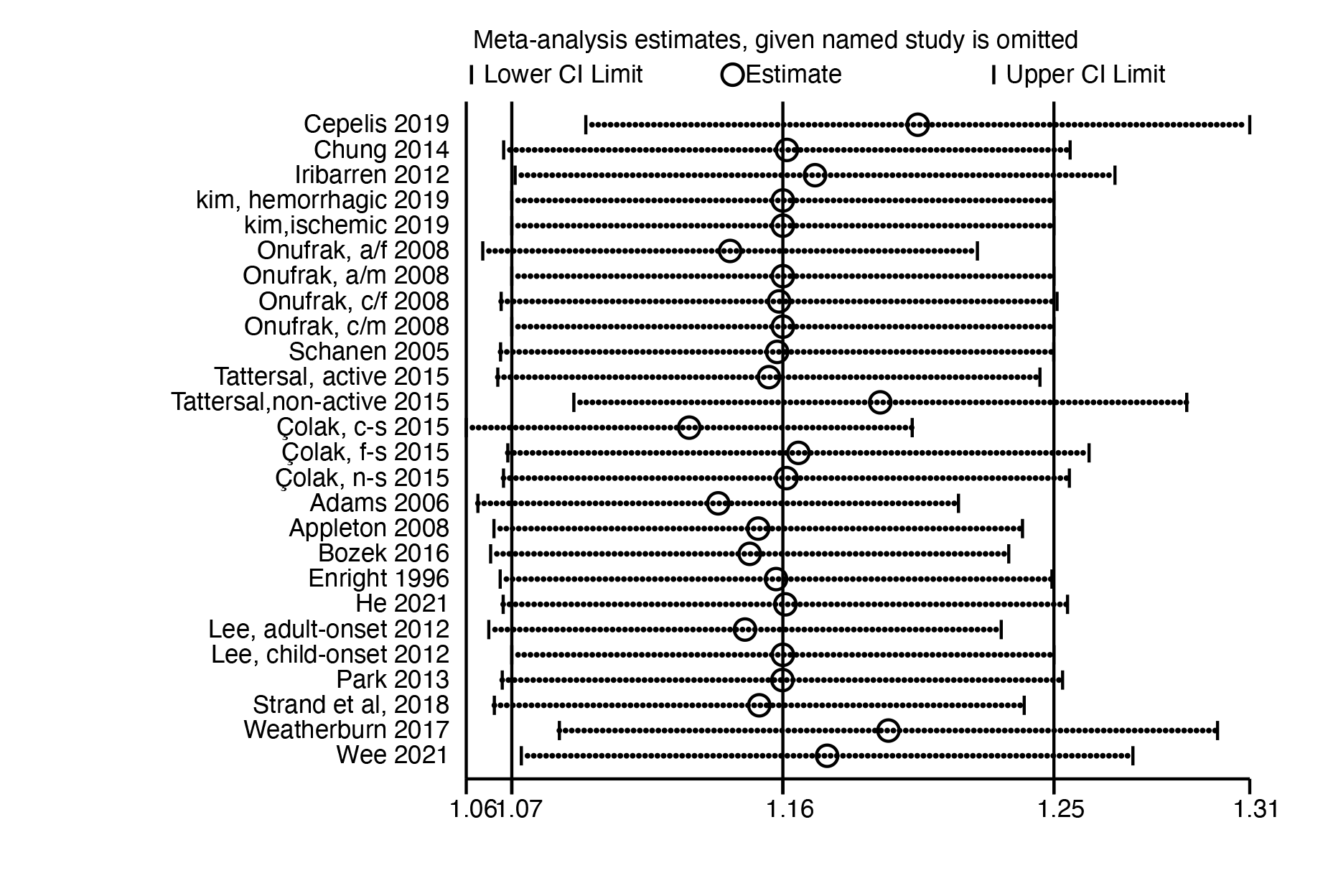

Supplement: Supplementary Figure 1 — Sensitivity analysis of association between asthma and the risk of stroke. CI, confidential interval; a/f, adult female; a/m, adult male; c/f, child female; c/m, child male; c-s, current smoking; f-s, former smoking; n-s, never smoking. [file Image_1.TIF]

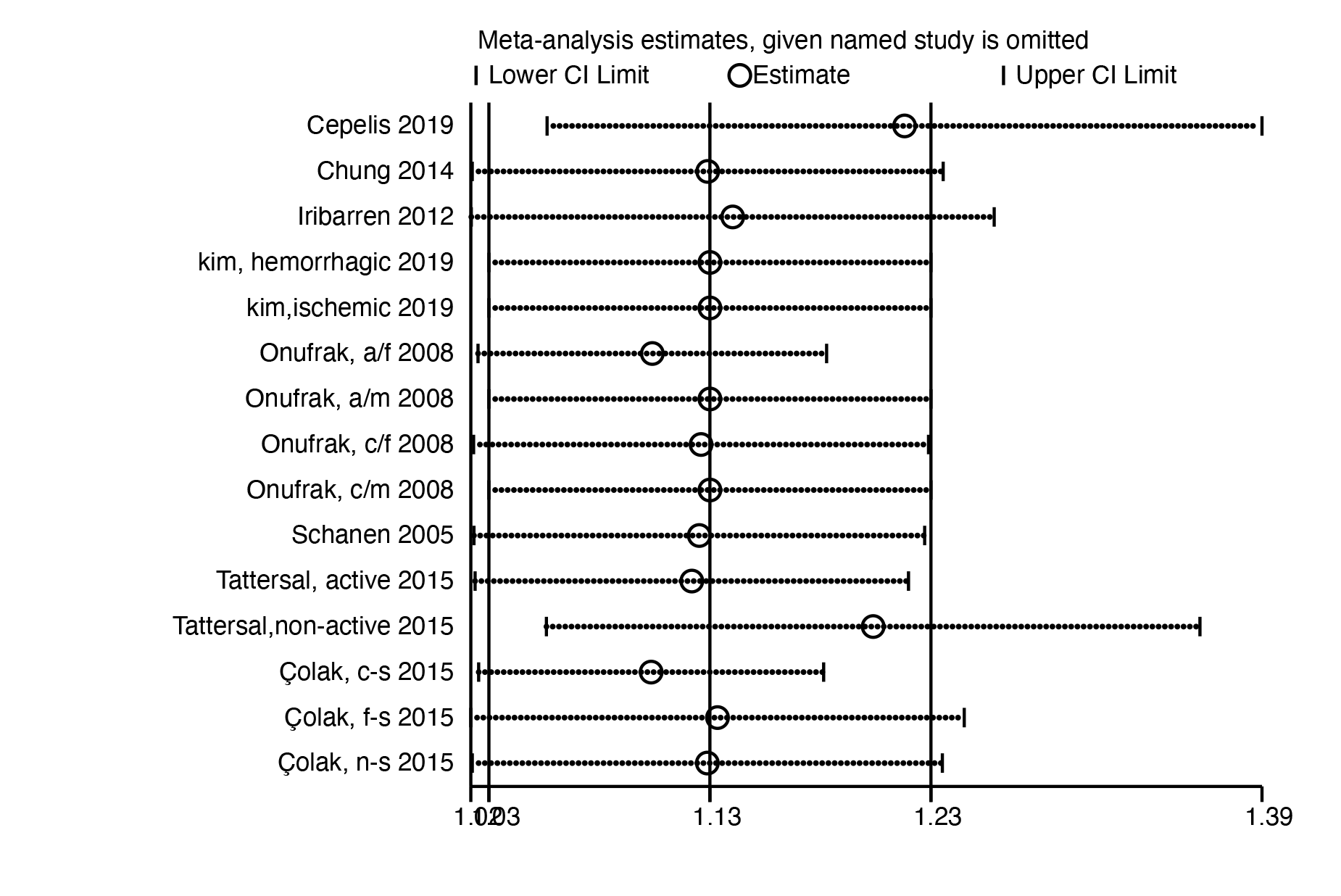

Supplement: Supplementary Figure 2 — Sensitivity analysis of association between asthma and the risk of stroke (only cohort studies included). CI, confidential interval; a/f, adult female; a/m, adult male; c/f, child female; c/m, child male; c-s, current smoking; f-s, former smoking; n-s, never smoking. [file Image_2.TIF]

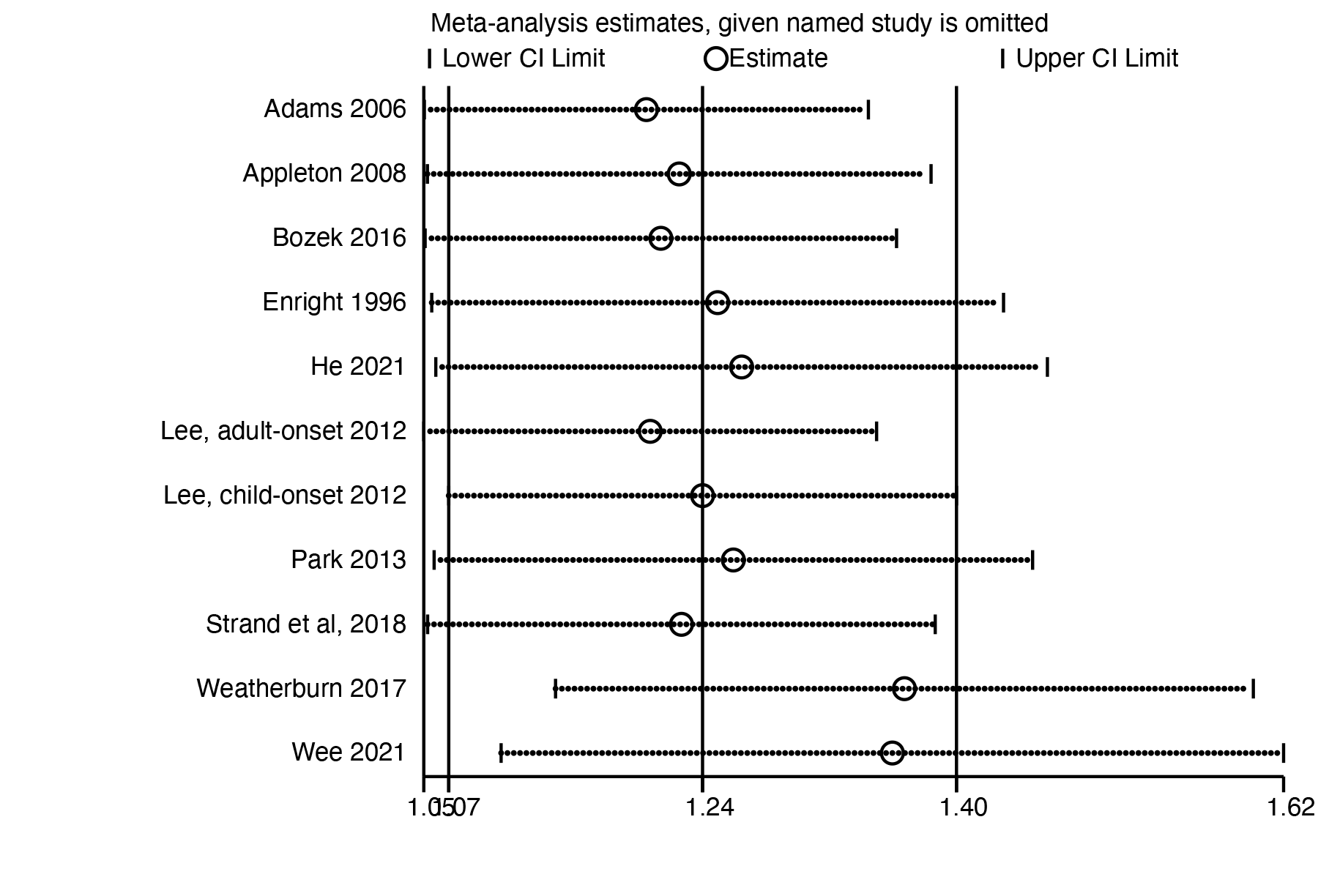

Supplement: Supplementary Figure 3 — Sensitivity analysis of association between asthma and the risk of stroke (only cross-sectional studies included). CI, confidential interval. [file Image_3.TIF]

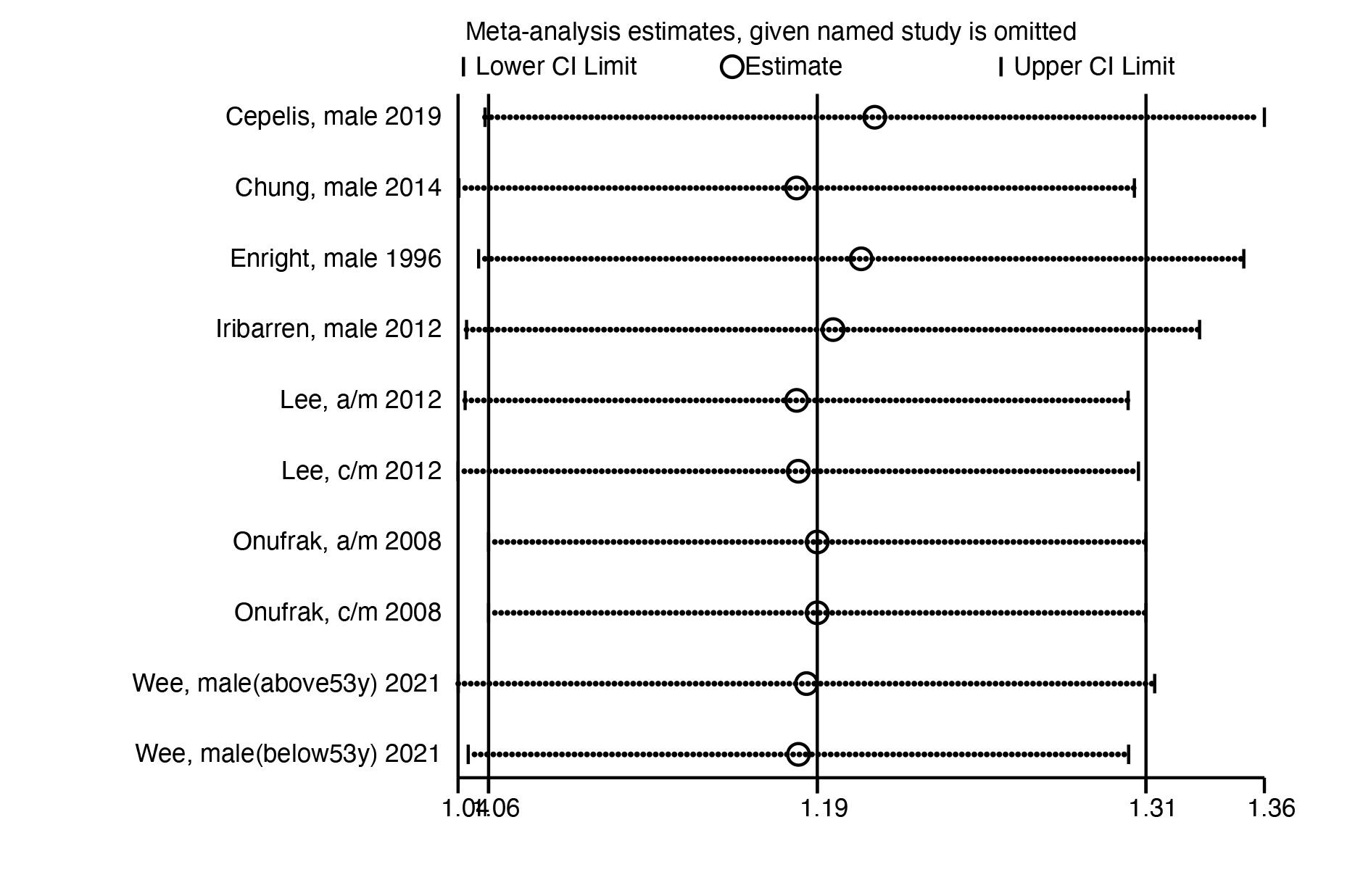

Supplement: Supplementary Figure 4 — Sensitivity analysis of association between asthma and the risk of stroke in male. CI, confidential interval; a/m, adult male; c/m, child male. [file Image_4.TIF]

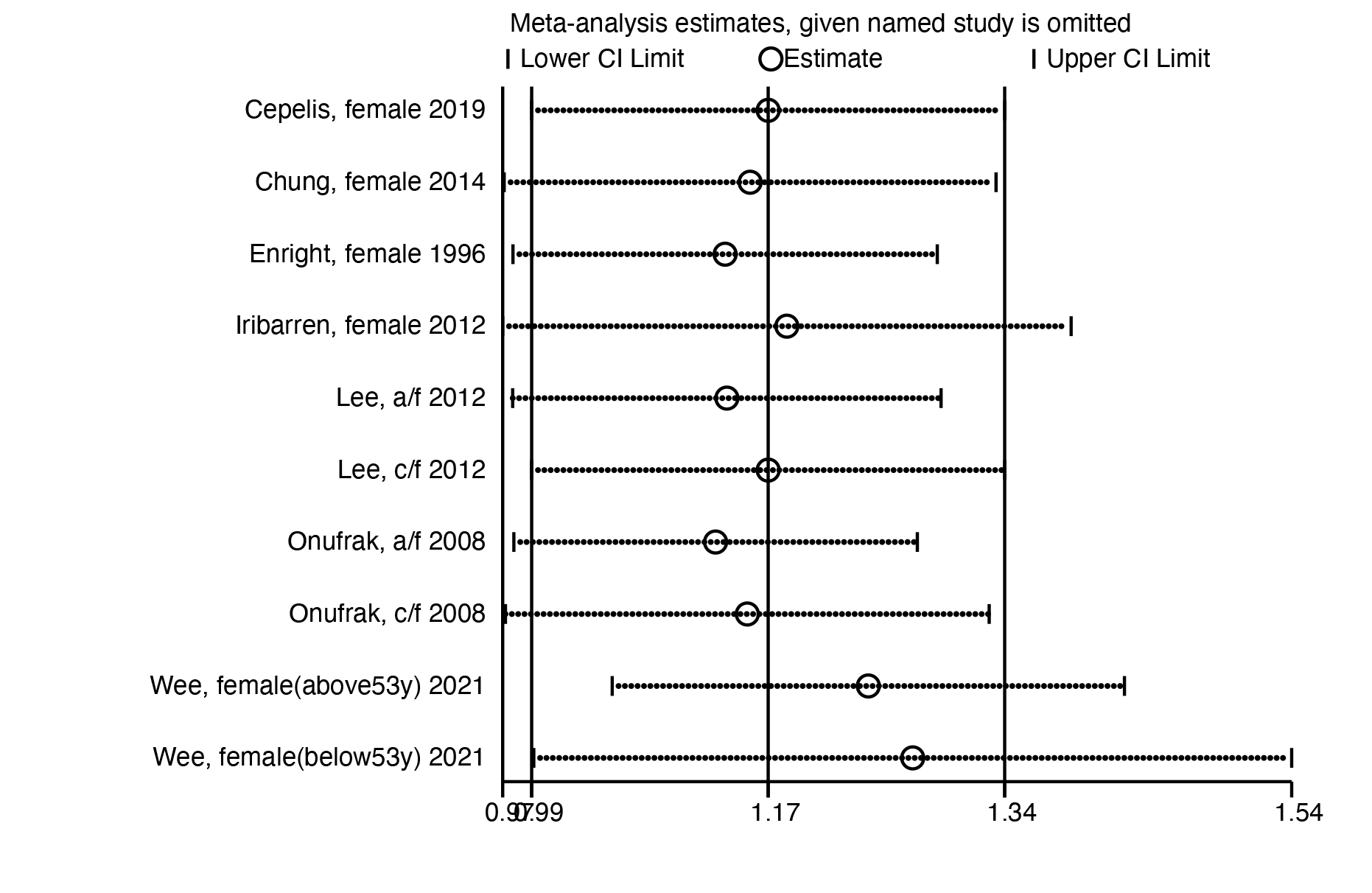

Supplement: Supplementary Figure 5 — Sensitivity analysis of association between asthma and the risk of stroke in female. CI, confidential interval; a/f, adult female; c/f, child female. [file Image_5.TIF]

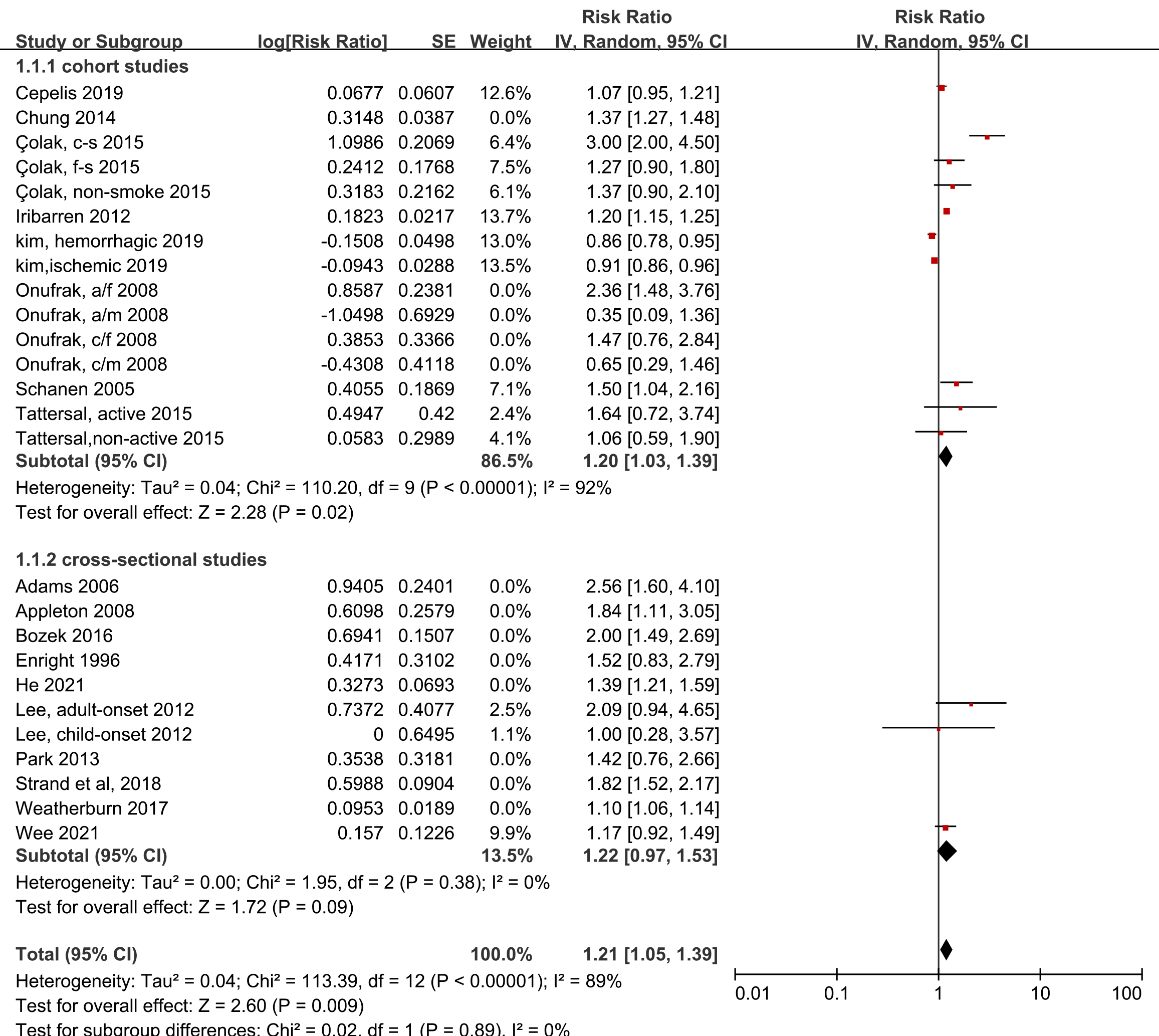

Supplement: Supplementary Figure 6 — Association between asthma and the risk of stroke (studies which did not adjust blood lipid, blood pressure, or diabetes mellitus were omitted). SE, standard error; IV, Inverse Variance method; df, degrees of freedom; CI, confidential interval; a/f, adult female; a/m, adult male; c/f, child female; c/m, child male; c-s, current smoking; f-s, former smoking; n-s, never smoking. [file Image_6.TIF]

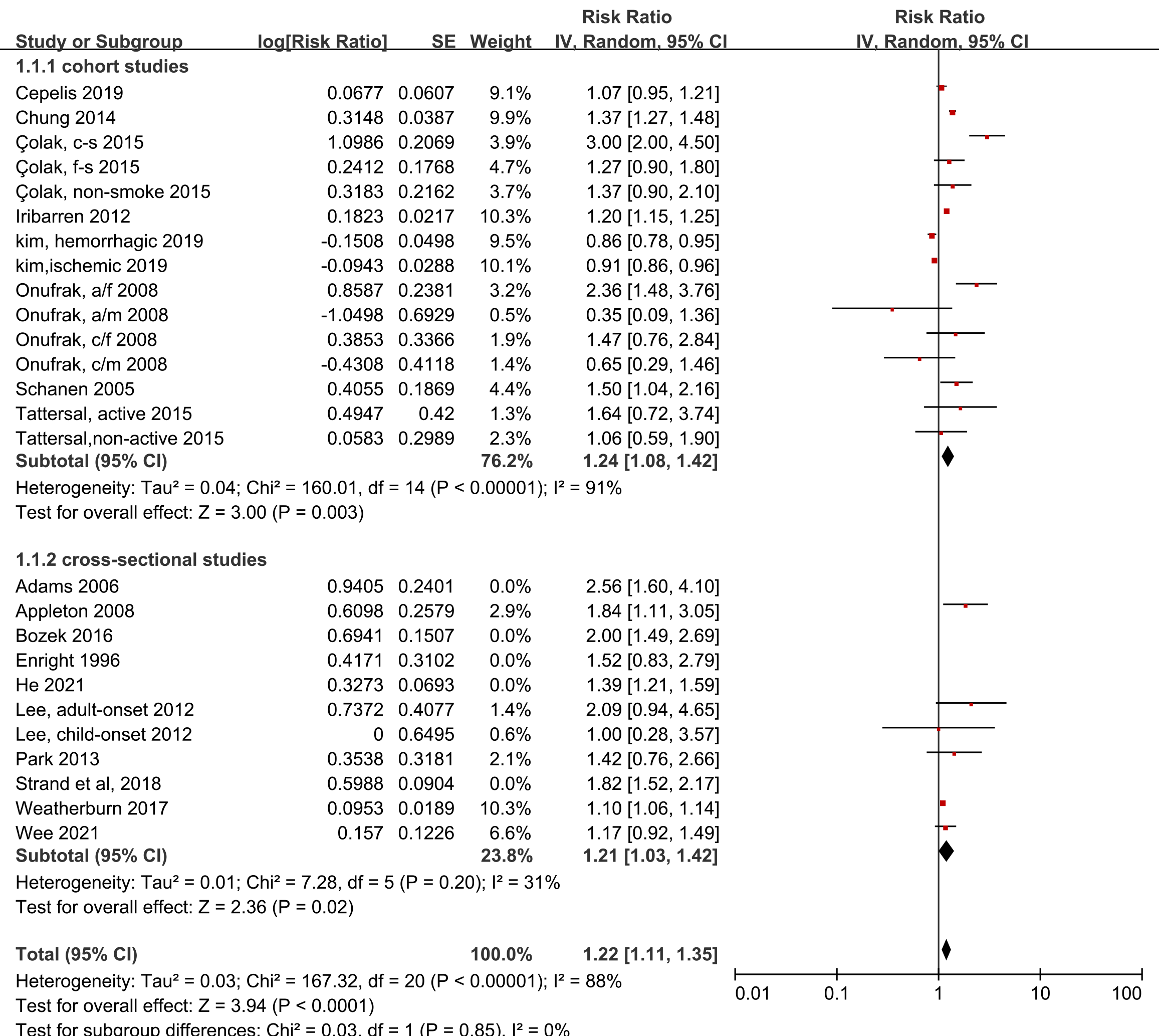

Supplement: Supplementary Figure 7 — Association between asthma and the risk of stroke (Two studies with only an association between asthma and stroke but not have the study outcomes and 4 low quailty studies were omitted). SE, standard error; IV, Inverse Variance method; df, degrees of freedom; CI, confidential interval; a/f, adult female; a/m, adult male; c/f, child female; c/m, child male; c-s, current smoking; f-s, former smoking; n-s, never smoking. [file Image_7.TIF]

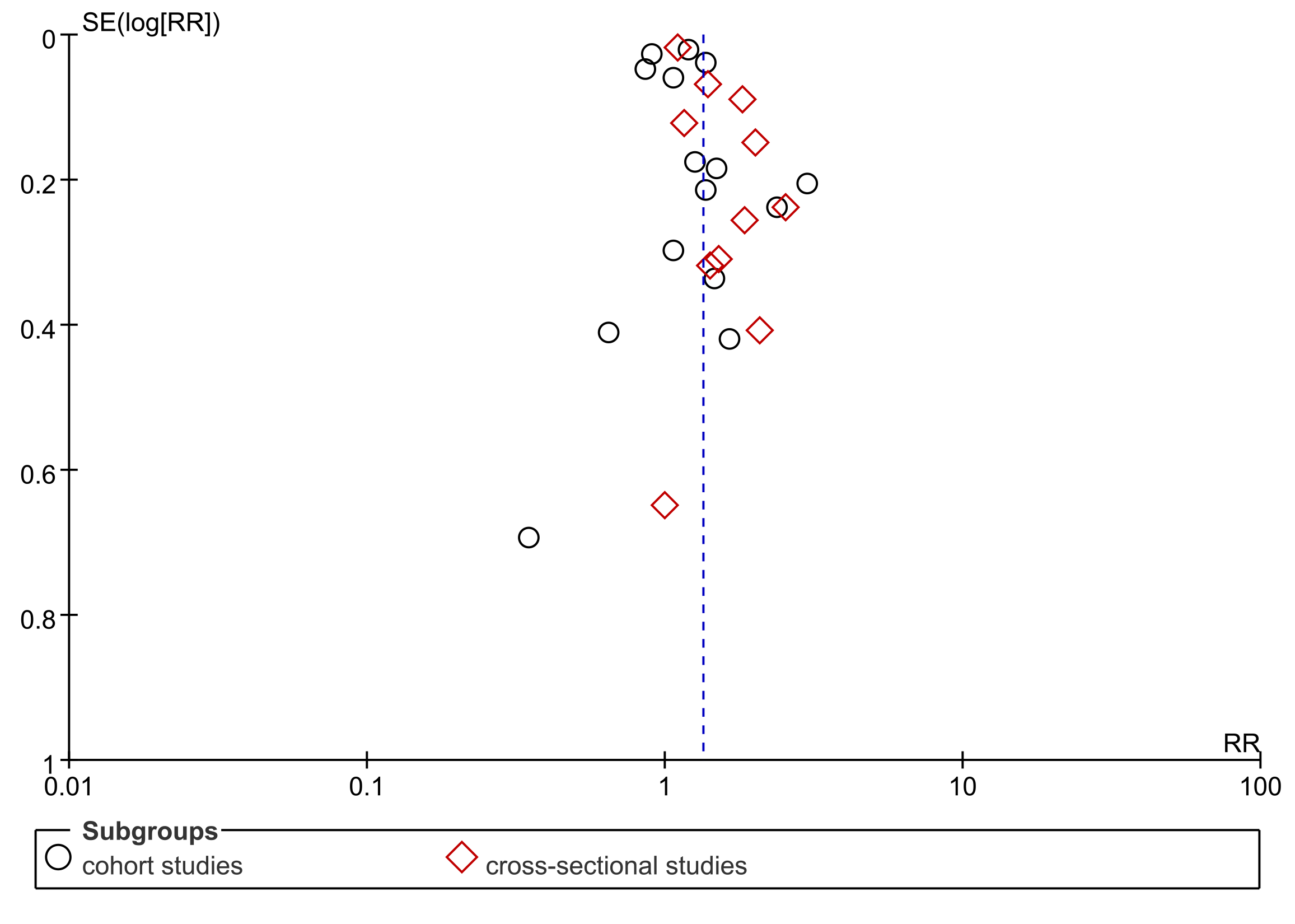

Supplement: Supplementary Figure 8 — Funnel plot depicting publication bias for the association between asthma and the risk of stroke. SE, standard error; RR, risk ratio. [file Image_8.TIF]
