# Supplementary material for: Meta-Analysis of the Association Between Asthma and the Risk of Stroke
Source: Front Neurol. 2022 Jun 24;13:900438. doi: 10.3389/fneur.2022.900438 (PMC9263265; doi:10.3389/fneur.2022.900438)
Supplement: Supplementary Table 2 — Search strategies. [file Table_2.docx]

| Supplementary Table 2 | |
| --- | --- |
| Search Strategies | |
| PubMed |  |
| Search number | Query |
| #1 | cerebrovascular accident*[Text Word] |
| #2 | cerebrovascular disease*[Text Word] |
| #3 | cerebral ischemia[Text Word] |
| #4 | cerebral hemorrhage[Text Word] |
| #5 | ischemic stroke*[Text Word] |
| #6 | hemorrhagic stroke*[Text Word] |
| #7 | transient ischemic attack*[Text Word] |
| #8 | apoplexy[Text Word] |
| #9 | CVA? [Text Word] |
| #10 | brain vascular accident*[Text Word] |
| #11 | embolic stroke*[Text Word] |
| #12 | thrombotic stroke*[Text Word] |
| #13 | brain infraction*[Text Word] |
| #14 | cerebrovascular disorder*[Text Word] |
| #15 | stroke[Mesh] |
| #16 | cerebrovascular disorders[Mesh] |
| #17 | #1 OR #2 OR #3 OR #4 OR #5 OR #6 OR #7 OR #8 OR #9 OR #10 OR #11 OR #12 OR #13 OR #14 OR #15 OR #16 |
| #18 | allergic airway inflammation[title] |
| #19 | asthma*[title] |
| #20 | asthmatic[title] |
| #21 | asthmaticus[title] |
| #22 | asthma[Mesh] |
| #23 | #18 OR #19 OR #20 OR #21 OR #22 |
| #24 | epidemiologic studies[Mesh] |
| #25 | cohort studies[Mesh] |
| #26 | epidemiologic[Text Word] |
| #27 | longitudinal[Text Word] |
| #28 | cohort[Text Word] |
| #29 | ‘follow up’[Text Word] |
| #30 | observational[Text Word] |
| #31 | prospective[Text Word] |
| #32 | retrospective[Text Word] |
| #33 | cross-sectional[text word] |
| #34 | cross-sectional studies[Mesh] |
| #35 | #18 OR #19 OR #20 OR #21 OR #22 OR #23 OR #24 OR #25 OR #26 OR #27 OR #28 OR #29 OR #30 OR #31 OR #32 OR #33 OR #34 |
| #36 | #17 AND #23 AND #35 |
| Embase |  |
| #1 | stroke:ti,ab,kw |
| #2 | 'cerebrovascular disease':ti,ab,kw |
| #3 | 'cerebrovascular accident':ti,ab,kw |
| #4 | 'cerebrovascular disorder':ti,ab,kw |
| #5 | 'brain ischemia':ti,ab,kw |
| #6 | 'brain hemorrhage':ti,ab,kw |
| #7 | 'brain vascular accident':ti,ab,kw |
| #8 | apoplexy:ti,ab,kw |
| #9 | 'transient ischemic attack':ti,ab,kw |
| #10 | 'cerebral ischemia':ti,ab,kw |
| #11 | 'cerebral hemorrhage':ti,ab,kw |
| #12 | 'stroke'/exp |
| #13 | #1 OR #2 OR #3 OR #4 OR #5 OR #6 OR #7 OR #8 OR #9 OR #10 OR #11 OR #12 |
| #14 | asthma:ti |
| #15 | asthmatic:ti |
| #16 | 'allergic airway inflammation':ti |
| #17 | asthmaticus:ti |
| #18 | #14 OR #15 OR #16 OR #17 |
| #19 | cohort |
| #20 | observational |
| #21 | prospective |
| #22 | retrospective |
| #23 | 'follow up' |
| #24 | longitudinal |
| #25 | cross |
| #26 | ‘cohort analysis’/exp |
| #27 | 'cross-sectional study'/exp |
| #28 | #19 OR #20 OR #21 OR #22 OR #23 OR #24 OR #25 OR #26 OR #27 |
| #24 | #13 AND #18 AND #28 |
| Web of Science |  |
| #1 | TI=(stroke) |
| #2 | TI=(cerebrovascular accident) |
| #3 | TI=(cerebrovascular disease) |
| #4 | TI=(cerebral ischemia) |
| #5 | TI=(cerebral hemorrhage) |
| #6 | TI=(transient ischemic attack) |
| #7 | #1 OR #2 OR #3 OR #4 OR #5 OR #6 |
| #8 | asthma |
| #9 | asthmatic |
| #10 | asthmaticus |
| #11 | allergic airway inflammation |
| #12 | #8 OR #9 OR #10 OR #11 |
| #13 | #7 AND #12 |
