# Supplementary material for: Meta-Analysis of the Association Between Asthma and the Risk of Stroke
Source: Front Neurol. 2022 Jun 24;13:900438. doi: 10.3389/fneur.2022.900438 (PMC9263265; doi:10.3389/fneur.2022.900438)
Supplement: Supplementary Table 3 — Newcastle-Ottawa scale for assessing the quality of included studies. [file Table_3.DOCX]

| **Supplementary Table 3** | | | | |
| --- | --- | --- | --- | --- |
| **Newcastle-Ottawa Scale for Assessing the Quality of Included Studies** | | | | |
| **Author, Year** | **Selection (max=4)** | **Comparability (max=2)** | **Exposure/Outcome (max=3)** | **Overall quality score (max=9)** |
| Onufrak et al. 2008 | **3** | **1** | **3** | **7** |
| Chung et al. 2014 | **3** | **1** | **3** | **7** |
| Çolak et al. 2015 | **3** | **1** | **3** | **7** |
| kim et al. 2019 | **4** | **1** | **3** | **8** |
| Cepelis et al. 2019 | **4** | **2** | **2** | **8** |
| Iribarren et al. 2012 | **3** | **2** | **2** | **7** |
| Schanen et al. 2005 | **4** | **2** | **2** | **8** |
| Tattersal et al. 2015 | **3** | **2** | **3** | **8** |
| Wee et al. 2021 | **3** | **2** | **2** | **7** |
| Enright et al. 1996 | **2** | **1** | **2** | **5** |
| Lee et al. 2012 | **3** | **1** | **2** | **6** |
| Adams et al. 2006 | **3** | **1** | **1** | **5** |
| Appleton et al. 2008 | **3** | **2** | **1** | **6** |
| Bozek et al. 2016 | **2** | **1** | **2** | **5** |
| Park et al. 2013 | **3** | **1** | **2** | **6** |
| Weatherburn et al. 2017 | **3** | **1** | **2** | **6** |
| Strand et al. 2018 | **3** | **1** | **1** | **5** |
| He et al. 2021 | **3** | **1** | **2** | **6** |
